# Denosumab: A Useful Addition to the Armamentarium for the Management of Male Osteoporosis

**DOI:** 10.7759/cureus.62736

**Published:** 2024-06-19

**Authors:** Jijith Krishnan, Sham Santhanam, Bhuwan Singh, Salim Patel, Divya G Bhojwani, Sameer Muchhala

**Affiliations:** 1 Medicine and Rheumatology, Government Medical College, Thrissur, Thrissur, IND; 2 Rheumatology, Kauvery Hospital, Chennai, IND; 3 Orthopedics and Traumatology, Mahaveer Hospital and Ortho Care Centre, Saharsa, IND; 4 Orthopedics and Traumatology, Dr. Kamdar's Nursing Home, Mumbai, IND; 5 Medical Affairs, Zydus Healthcare Limited, Mumbai, IND

**Keywords:** trauma, osteoporosis, bone mineral density, monoclonal antibodies, bisphosphonate, denosumab

## Abstract

Bone is a dynamic tissue. It remodels, preserving serum calcium, repairing microdamage, and maintaining strength. Osteoporosis is caused by a decrease in bone strength, which manifests clinically as low-energy vertebral and non-vertebral fractures. Osteoporosis poses a significant public health challenge. While it's often portrayed as primarily impacting postmenopausal women, there's been growing recognition among researchers and clinicians regarding its prevalence in men. Major fracture in men has higher mortality rates than in women. Denosumab is a fully human monoclonal immunoglobulin G2 (IgG2) antibody that binds to RANKL, the principal regulator of osteoclastic bone resorption. Multiple studies suggest that denosumab is both effective and safe, exhibiting higher adherence rates and greater patient satisfaction. In this narrative review, we highlighted the effects of denosumab in men with osteoporosis, subsequent changes in bone mineral density, and bone turnover markers outlining the literature and guideline support.

## Introduction and background

Osteoporosis, identified as a widespread inflammatory bone loss condition, is intricately linked to the aging process, with its prevalence increasing proportionally with age. This pattern is further intensified by the continual aging of the population [1]. Within the confines of India, a nation accommodating a populace surpassing 1.3 billion individuals, it is noteworthy that around 230 million citizens have reached the age of 50 and beyond [2]. In the Global Burden of Disease 2019 study, India ranks first amongst analyzed 204 countries and territories with the highest disability-adjusted life-years (DALYs) burden and death due to low bone mineral density (BMD)-related fractures [3]. A study conducted across India identified that osteoporosis and osteopenia affect one out of five and one out of two adults, respectively. Also, two out of five elderly women and one out of three elderly men have identified with osteoporosis [4].

Male osteoporosis is a pathological condition that is still mostly undiagnosed and untreated, primarily because screening is done infrequently and because BMD testing criteria are controversial [5]. As many patients who sustained fragility fractures did not report a BMD impairment, the prevalence of osteoporosis in men is believed to be lower than it actually is. In general, the frequency of fractures in men reaches a bimodal peak between the ages of 18 years and 45 years (traumatic fractures) and between the ages of 75 years and 80 years (osteoporotic fractures). Although the prevalence of osteoporosis is substantially higher in women, men account for between 30% and 40% of all osteoporotic fractures [5].

An occurrence of a vertebral or femoral fracture is projected to happen every 200 seconds, accompanied by a mortality rate ranging from 15% to 25%. Osteoporosis and osteoporotic fractures are significant public health concerns for both men and women globally, but men are underrepresented in osteoporosis trials, clinical and scientific evidence of osteoporosis in males, as well as variations in the effectiveness and adverse effects of anti-osteoporotic medications, are not well comprehended [1]. This review focuses on denosumab use in male osteoporosis succinctly and aims to summarize all the available studies and the impact of denosumab therapy mitigating fracture risk, supplemented by clinical insights on denosumab utilization.

Pathogenesis of osteoporosis in men

Men develop osteoporosis as a result of a complex interplay of various variables that result in bone loss and microstructural disturbance. Estradiol and testosterone production declines in older men. Men experience slow bone and endure a lesser total decline in BMD than women do, who undergo an abrupt drop in estrogen levels after menopause that accelerates bone loss. Trabecular thinning is a sign of bone loss in men, whereas trabeculae are lost in women [6].

Risk factors and contributors to osteoporosis in men

The occurrence of osteoporosis in men has been linked to a number of risk factors [7]. Overall, the health of bones is influenced by factors such as age, lifestyle, hormones, genetics, coexisting conditions, and medical interventions (Table 1).

**Table 1:** Risk factors and contributors to osteoporosis in men Image Credit: Dr. Divya Bhojwani

Primary osteoporosis	Secondary osteoporosis
Age-related	Cushing syndrome
Idiopathic diseases	Excessive alcohol intake
	Corticosteroid therapy
	Primary or secondary hypogonadism
	Low calcium intake
	Smoking
	Vitamin D deficiency or insufficiency
	Family history of minimal trauma fracture
	Low body mass index
	Some antiepileptic medications
	Thyrotoxicosis or thyroxine over-replacement
	Chronic kidney or liver disease
	Primary hyperparathyroidism
	Rheumatoid arthritis
	Ankylosing spondylitis
	Diabetes mellitus (both type 1 and type 2)
	Chronic obstructive pulmonary disease
	Osteogenesis imperfecta

Clinical indications for initiating therapy

Table 2 summarizes key indications for initiating anti-osteoporotic therapy in adults as recommended by The Indian Society of Bone and Mineral Research (ISBMR) [2].

**Table 2:** Key indications for initiating anti-osteoporotic therapy Image Credit: Dr. Divya Bhojwani DXA: dual-energy X-ray absorptiometry; FRAX: fracture risk assessment tool

Sr. No.	Key indication for initiating anti-osteoporotic therapy
1	A fracture in the vertebrae, whether clinically evident or detected through vertebral imaging, or a non-vertebral fracture involving the hip, wrist, or humerus.
2	In individuals aged over 50 with a T-score equal to or less than -2.5 at the femoral neck, total hip, or lumbar spine as measured by DXA.
3	In individuals diagnosed with osteopenia (T-score ranging from -1.0 to -2.5 at the femoral neck or lumbar spine) who present clinical risk factors or exhibit a 10-year probability of a hip fracture equal to or exceeding 3.5%, or a 10-year probability of a major osteoporosis-related fracture equal to or exceeding 10.5% as assessed by the FRAX tool (with limited data available for Indians).
4	For individuals with type 2 diabetes mellitus, the intervention threshold should be raised to a T-score equal to or less than -2.0 at the femoral neck, total hip, or lumbar spine as measured by DXA.

Overview of current management of osteoporosis in men

Clinical examination, fracture risk assessment, diagnostic workup, and BMD measurements should all be taken into consideration when deciding on a treatment plan for males with osteoporosis [7]. Initiating osteoporosis therapy at the earliest opportunity is crucial to alleviate the burden of fragility fractures, mitigating the associated morbidity, mortality, and expenses. There is strong evidence that those who present with hip or spine fractures will see a substantial decrease in their risk of suffering another fracture in the future if they receive the proper osteoporosis treatment [2].

Fundamentals of Osteoporosis Management

Men who have osteoporosis or are at risk for developing it are advised to get adequate amounts of vitamin D and calcium through diet (and supplementation, if necessary). Supplementation with calcium and vitamin D has been shown to moderately decrease bone loss in the femoral neck, spine, and whole body, presenting a reduced risk of non-vertebral fractures in individuals aged 65 and older, both in men and women [7].

The American College of Physicians recommends administering pharmacologic therapy to individuals with confirmed osteoporosis and those who have experienced fragility fractures in the past. Meanwhile, the Endocrine Society suggests pharmacological therapy for men at a high risk of fractures. Currently, available osteoporosis therapies for men in India encompass antiresorptive such as alendronate, ibandronate, risedronate, and zoledronic acid, anabolic agent teriparatide, and a fully-humanized monoclonal antibody RANKL inhibitor denosumab [7].

Due to the increased likelihood of experiencing adverse events, including cardiac incidents and breast cancer (though the risk of breast cancer is not elevated when using estrogen alone), hormone replacement therapy (HRT) is not advised for managing osteoporosis, despite being effective in increasing bone mass and preventing fractures [2]. Bisphosphonates are most commonly used as first-line therapy. Oral administration of risedronate and alendronate is often done on a daily or weekly basis. Once a year, zoledronic acid is intravenously injected [7]. Teriparatide is an effective anabolic agent to initiate therapy in these cases, which is to be continued for 24 months and followed by antiresorptives [2].

Denosumab: A Unique Mechanism of Action

Denosumab is a fully human monoclonal immunoglobulin G2 (IgG2) antibody that binds to RANKL, the principal regulator of osteoclastic bone resorption. Thus, it prevents RANKL from binding to the RANK receptor located on the osteoclast precursor cells and osteoclasts. Osteoblasts produce RANKL, a member of the tumor necrosis factor protein family. It is a soluble protein responsible for encouraging bone resorption by stimulating a signaling cascade in osteoclasts. Osteoclast development, activity, and survival are inhibited by denosumab's high-affinity binding to RANKL. This interaction leads to enhanced cortical and trabecular bone mass and strength. Denosumab is administered by subcutaneous injection of 60 mg every six months in the upper arm, upper thigh, or abdomen [8]. With a half-life of approximately 26 days, it circulates in the bloodstream, binds to RANKL in the extracellular fluid, and is eliminated through the reticuloendothelial system [9]. Denosumab does not reside in the skeleton and has a biological effect that lasts as long as it is in systemic circulation because of its distinct method of action in the extracellular environment [8].

## Review

Literature search and study selection criteria

**Figure 1:**
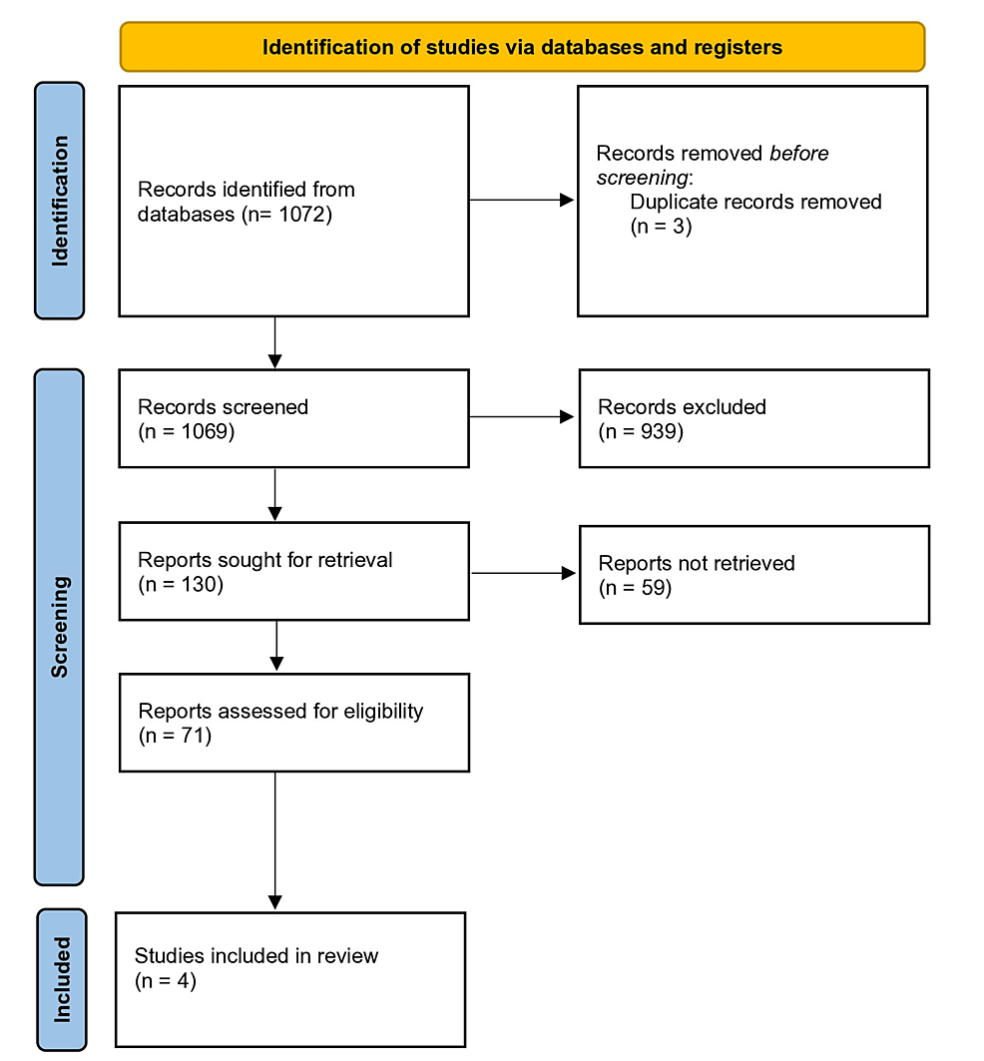
PRISMA flow diagram of study screening and selection PRISMA: Preferred Reporting Items for Systematic Reviews and Meta-Analysis

A search strategy employing keywords was utilized to explore databases for the retrieval of study abstracts. The databases PubMed and ClinicalTrials. gov was used with various combinations of keywords such as "osteoporosis," "denosumab," "male osteoporosis" and “male” until December 2023 in the English language only. The search strategy resulted in 1072 abstracts. Studies that evaluated change in BMD, reduction in fracture risk, and bone turnover markers post-denosumab use compared to placebo, bisphosphonates, or anabolic therapies in patients with male osteoporosis were included in this review. All four of these studies were included in the final analysis while studies discussing the use of denosumab in post-menopausal women, glucocorticoid-induced osteoporosis, denosumab discontinuation, and prostate cancer were excluded. The titles, abstracts, and headings of the articles retrieved on database search were screened to exclude duplicate publications and irrelevant studies. Quantitative synthesis was not conducted due to inconsistencies in the reporting of data across all studies. The PRISMA (Preferred Reporting Items for Systematic Reviews and Meta-Analyses) flowchart is depicted in Figure 1.

Results

Bone Mineral Density

Data on the impact of denosumab therapy on BMD were reported in three studies [9-12]. After randomized controlled trials (RCTs) in postmenopausal women showed promising findings, Orwoll et al. conducted a phase III multicenter, randomized, double-blinded, placebo-controlled study - ADAMO. This landmark study was conducted to evaluate the effectiveness and safety of denosumab in males with low BMD upon which the U.S. Food and Drug Administration (FDA) and European Medicines Agency (EMA) extended the use of denosumab in men at increased risk of fractures. The primary endpoint was the percentage change from the initial measurement in lumbar spine (LS) BMD at the 12-month mark. In this study, 242 men aged 30-85 diagnosed with osteoporosis were randomly assigned to either receive denosumab or a placebo (with 121 participants in each group). After 12 months, denosumab demonstrated a significant increase in BMD, registering at 5.7% in the LS, 2.4% in the total hip, 2.1% in the femoral neck, 3.1% at the trochanter and 0.6% at the one-third radius (adjusted p ≤ 0.0144 for BMD percentage differences at all sites compared with placebo). The authors also reported that the denosumab treatment remained unaffected by baseline BMD T-score, estimated 10-year fracture risk, C-terminal telopeptide of type 1 collagen (sCTX), testosterone levels, age, race, geographic region, or prior osteoporotic fractures [9]. In a retrospective analysis by Jeong et al., 147 Korean male osteoporosis patients who received denosumab were evaluated. Following a 12-month treatment period, 60 patients were lost to follow-up, and 8 individuals were excluded from the final analysis due to missing data. Further analysis was performed based on the patient’s history of anti-osteoporotic medication. They found that in treatment-naïve patients (n=54), there was a significant increase in BMD at all sites: 5.2% in the LS, 2.3% in the femoral neck, and 1.9% in the total hip (p<0.01) compared to the initial baseline. In the group previously treated with bisphosphonates (n=25), the BMD increased significantly at the LS (4.8%, p<0.01); however, the increased BMD at the femoral neck (1.4%, p=0.06) and total hip (0.8%, p=0.06) was not significant. The results of the study conducted by Jeong et al. were consistent with the findings of the ADAMO trial. The increase in BMD at LS was at a similar percentage as that of the ADAMO trial (around 5.7%) [11]. Osteoporosis and skeletal fractures, especially below the level of the damage, arise after a spinal cord injury (SCI) due to the significant loss of bone mass and increase in remodeling. One year after SCI, more than 50% of patients with complete SCI develop densitometric osteoporosis, which can reach 81% of individuals after more than five years of SCI. Gifre et al. studied the impact of anti-osteoporotic therapy on osteoporosis linked to SCI. They reported the effect of denosumab treatment on BMD in individuals with SCI who developed osteoporosis during follow-up and received denosumab therapy for 24 months. After 12 months of denosumab therapy, patients had a significant increase in BMD at the LS (+7.47%, p=0.001) and the femoral neck (+3.03%, p=0.019), with a cumulative increase after 24 months of follow-up with (+9.1%, p=0.002) at LS and (+4.4%, p=0.033) at the femoral neck. Hence, the administration of denosumab over 24 months was linked to a continuous and substantial augmentation in bone mass throughout all skeletal sites. This encompassed the LS and sub-lesional level, as well as the proximal femur [12].

Fractures

Amongst the studies reviewed, only three studies reported data on fractures [9,10,12]. Each study employed a slightly different protocol for evaluating changes in BMD within the control and treatment groups. Moreover, each study utilized somewhat varied methods for presenting their findings. Consequently, rather than undergoing quantitative analysis, the results of each study will be reported individually. Orwoll et al. found that clinical fractures occurred in 1.7% (n=2) and 0.8% (n=1) in the placebo and denosumab treatment arms, respectively [9]. Silverman et al. while evaluating the cost-effectiveness of denosumab reported that patients receiving denosumab had the lowest 10-year chances of hip fractures in the model (lifetime cohort Markov model) compared to those receiving all other treatments (Alendronate, Zoledronate, Risedronate, Ibandronate, and Teriparatide) [10]. Although the study conducted by Gifre et al. was an observational study that included a small number of patients, no new skeletal fractures were reported during the 24-month follow-up period post-denosumab use [12].

Bone Turnover Markers (BTM)

Three studies reported a change in BTM after denosumab treatment [9,11,12]. All three studies evaluated changes in the C-terminal telopeptide of type 1 collagen (CTX) and procollagen 1 N-terminal propeptide (P1NP) at different points of time. Orwoll et al. reported treatment with denosumab decreased median sCTX concentration compared with placebo at day 15 (adjusted p<0.0001). At day 15, the median percent decrease in sCTX concentration from baseline was -7% in the placebo group and -81% in the group receiving denosumab [9]. Jeong et al. in Korean male patients after 12 months of treatment reported noteworthy reductions, including a decrease of -55.1% in CTX and -62.9% in P1NP among drug-naïve patients (p<0.01). Additionally, patients with prior bisphosphonate treatment demonstrated declines of -37.7% in CTX and -55.4% in P1NP (p<0.01). The authors also reported that the BTM showed a rapid decline in the first six months and continued to significantly decline over the following 12 months in both groups [11]. Gifre et al. also studied and reported the improvement in BTM. At 12 months of denosumab treatment, a significant decrease in BTM such as -41% bone FA, -53% P1NP, and -59% CTX was reported. With the additional increase in BMD at 24 months of treatment and follow-up, a further overall decrease in BTMs was reported by the authors (-38% bone FA, -43% P1NP, and -42% CTX, respectively) [12].

Adverse Events

Adverse events/side effects were reported in three studies. Orwoll et al. reported overall the incidence of adverse events, serious adverse events, and fatal adverse events was comparable between treatment groups. The most frequent adverse events reported were back pain, nasopharyngitis, constipation, and arthralgia which were mild or moderate. The authors also reported that there were no complications of fracture healing, osteonecrosis of the jaw, hypocalcemia, and atypical femoral fractures observed during the 12-month follow-up period. It is advised to recheck calcium levels within two weeks of the first dose in patients who are predisposed to hypocalcemia and regular monitoring every six months to check on the adverse events. Among the serious adverse events, prostate cancer was documented in three men (2.5%) within the denosumab group, with none reported in the placebo group. Notably, two of the three cases were diagnosed within three weeks of the initial denosumab dose, indicating pre-existing cancer before treatment initiation. Arterial limb thrombosis serious adverse events occurred in two men (1.7%) in the denosumab group, with none in the placebo group. During the 12-month double-blind treatment period, two fatalities were reported - one in the denosumab group resulting from myocardial infarction and one in the placebo group due to basilar artery thrombosis. Importantly, neither death was deemed to be related to the treatment [9]. Jeong et al. also reported there was no significant change in the calcium levels during the treatment. No serious adverse events such as severe hypocalcemia, osteonecrosis of the jaw, fracture healing complications, or atypical femoral fracture were reported [11]. Gifre et al. observed no side effects related to denosumab treatment during the 24-month follow-up [12].

Table 3 shows a concise overview of the published literature on denosumab included in the review and its effects on BMD, fracture risk, bone turnover markers, and adverse events.

**Table 3:** Summary of published literature about the use of denosumab in the management of male osteoporosis BMD: bone mineral density; LS: lumbar spine; CTX: C-terminal telopeptide; P1NP: procollagen type I N-terminal propeptide

Author	Year	Subject (n = total number of subjects enrolled)	Study outcome
Orwoll et al. [9]	2012	N = 242	After 12 months of therapy, denosumab resulted in increased BMD - 5.7% at the LS, 2.1% at the femoral neck, 2.4% at the total hip, 0.6% at the one-third radius, 3.1% at the trochanter, and (adjusted p ≤ 0.0144 for BMD percent differences at all sites were comparable with placebo). Significantly reduced serum CTX levels at d 15 after denosumab treatment (adjusted p < 0.0001). Adverse events were similar in both the denosumab and placebo groups.
Silverman et al. [10]	2015	Not mentioned (only elderly osteoporotic men ≥ 75 years)	Denosumab had the lowest 10-year chances of hip fractures compared to other osteoporotic treatments. Denosumab therapy for the elderly (osteoporotic men ≥ 75 years) is cost-effective compared to other therapies available (Alendronate, Zoledronate, Risedronate, Ibandronate, and Terparatide).
Jeong et al. [11]	2022	N = 79	A significant increase in BMD was observed in drug-naïve patients In patients with a history of bisphosphonate use, denosumab significantly increased BMD at LS. Bone turnover markers (CTX and P1NP) significantly decreased in both groups after 12 months of treatment.
Gifre et al. [12]	2022	N = 13	Significant increase in BMD at LS and proximal femur in patients with osteoporosis post spine cord injury after 12 months of denosumab treatment. Denosumab treatment over a span of 24 months demonstrated a continuous and notable increase in bone mass across all skeletal sites. Bone turnover markers significantly decreased at 12 months and remained decreased at the 24-month follow-up. No skeletal fractures and adverse events were observed during follow-up.

Discussion

In the context of osteoporosis, establishing a treatment goal serves to guide both the patient and physician in selecting an initial therapy to achieve the target. It provides insights into the patient's responsiveness to the chosen treatment and facilitates informed decision-making regarding whether to discontinue, maintain, or modify the treatment, contingent upon the progress made toward achieving the treatment target. Denosumab is becoming an increasingly utilized therapy in the management of osteoporosis due to its significant efficacy, tolerability, and convenient dosage. Its unique property of not residing in the skeleton exerting a biological impact that lasts as long as it is in systemic circulation further warrants its use in the management of osteoporosis especially in patients with high fracture risk [8,13].

Denosumab is the first and only RANKL inhibitor approved for the treatment of osteoporosis. It has reversible effects on discontinuation and is particularly effective at decreasing bone turnover. Since obtaining approval, it has consistently demonstrated prolonged efficacy in enhancing BMD and reducing the risk of fractures. Individual benefit-risk assessments should be made before the initiation of denosumab therapy in the management of osteoporosis.

The review of denosumab therapy across multiple studies reveals promising outcomes in terms of BMD, fractures, BTM, and adverse events. In particular, the ADAMO trial, a significant phase III study, demonstrated that denosumab effectively increased BMD in men with low BMD, influencing key skeletal sites such as the LS, total hip, femoral neck, trochanter, and one-third radius. This positive impact persisted across diverse patient characteristics, including baseline BMD, fracture risk, and prior osteoporotic fractures. Additionally, a retrospective analysis and an observational study reinforced the efficacy of denosumab in increasing BMD, with a notable reduction in BTMs over time [9,11]. All these findings of studies support the conclusion that denosumab 60 mg once in six months is similarly effective in the management of male osteoporosis as in post-menopausal women with osteoporosis.

Regarding fractures, while clinical fractures occurred in a limited number of cases, denosumab showcased its potential to reduce the 10-year chances of hip fractures. Notably, the observational study reported no new skeletal fractures during the 24-month follow-up post-denosumab use. Moreover, denosumab exhibited a favorable impact on BTMs, showcasing a consistent reduction in markers such as sCTX and P1NP across different studies. The improvement in BTMs was accompanied by a significant increase in BMD [9-12]. In the majority of men, bone loss primarily occurs as a consequence of an unfavorable remodeling balance characterized by diminished bone formation, frequently leading to trabecular thinning. Conversely, in women, bone loss is attributed to heightened bone resorption, resulting in trabecular separation. Interestingly, these studies showed the decreasing trend of BTM with denosumab decreasing the risk of fractures.

In terms of adverse events, denosumab demonstrated a comparable safety profile to placebo, with mild or moderate adverse events reported. Notably, serious adverse events were infrequent, and fatalities were not deemed treatment-related. Prostate cancer cases and arterial limb thrombosis, while reported in a small percentage, were not significantly associated with denosumab treatment [9,11,12].

Numerous systematic reviews and meta-analyses have been carried out, specifically addressing the use of denosumab in the treatment of postmenopausal women with osteoporosis or low BMD. The findings of the meta-analysis were consistent observing a significant decrease in bone markers, an increase in BMD, and a significant reduction in the risk of fracture after treatment with denosumab [13-16]. In a narrative review on denosumab in the treatment of osteoporosis after 10 years of denosumab treatment, maintaining bone turnover inhibition over a decade, exhibited a positive balance between benefits and risks [17]. It was observed that denosumab reduces the risk of vertebral fractures and has better efficacy in high-risk subgroups (with ≥2 risk factors) while better efficacy for subgroups with BMI < 25 kg/m^2^, hip and femoral neck BMD T score ≤ -2.5. Moreover, patients administered denosumab experienced a significant reduction in the risk of different fracture types, including clinical fractures, non-vertebral fractures, vertebral fractures, and hip fractures. Denosumab demonstrates comparable efficacy to other osteoporosis treatment agents, such as bisphosphonates [18]. The comparison of fractures prevented per skeletal adverse events, such as osteonecrosis of the jaw and atypical femoral fracture, revealed a favorable profile. Additionally, the occurrence of adverse events, including infections and malignancies, remained consistently low in the aging study population over the extended treatment period [17]. Moreover, it is considered safe for use in vulnerable populations, including patients with chronic kidney disease and individuals undergoing aromatase inhibitor treatment for breast cancer [18].

Table 4 further outlines the practice highlights to aid how clinicians can add denosumab in their existing armamentarium, monitor patients, safely transition away from denosumab, and guideline recommendations to further facilitate the management of osteoporosis.

**Table 4:** Practice highlights for clinicians USFDA: United States Food and Drug Administration; EMA: European Medicines Agency; DCGI: Drugs Controller General of India; ONJ: Osteonecrosis of the jaw; BMD: bone mineral density; ECTS: European Calcified Tissue Society; AACE: American Association of Clinical Endocrinologists/American College of Endocrinology

Drug name	Denosumab
Where can denosumab be used?	Denosumab is approved by USFDA, EMA, and DCGI to be used in the treatment of increasing bone mass in men with osteoporosis at a high risk of fractures, addressing bone loss associated with prolonged systemic glucocorticoid therapy in adult patients, enhancing bone mass in men at an increased risk of fracture undergoing androgen deprivation therapy for non-metastatic prostate cancer, promoting bone mass in women at a heightened risk of fracture receiving aromatase inhibitor therapy for breast cancer and post-menopausal osteoporosis [2,19].
Pre-administration checklist and screening	Calcium levels should be assessed before the initiation of denosumab. Hypocalcemia must be corrected before initiating therapy with denosumab^. ^Investigations to rule out secondary causes for osteoporosis in men (testosterone, ferritin, coeliac disease, etc.) [20].
Medication monitoring	Monitoring of serum creatinine, phosphorus, serum calcium and magnesium, signs and symptoms of hypocalcemia especially in patients with thyroid/parathyroid surgery, severe renal impairment, hypoparathyroidism and malabsorption syndromes, routine dental check-up if risk factors for ONJ and also monitor for signs of hypersensitivity [20]. For patients not suffering from advanced chronic kidney disease but who are at risk of hypocalcemia and mineral metabolism disorders (such as those undergoing treatment with other medications that reduce calcium levels), it is advisable to evaluate serum calcium and mineral concentrations (including phosphorus and magnesium) within 10 to 14 days following a denosumab injection [21].
Should Denosumab be used indefinitely?	Due to denosumab’s unique mechanism of action, it does not reside in the skeleton and has biological effects that last as long as it is in systemic circulation. It is also well tolerated and safe, with adverse events being rare.The full reversibility of denosumab supports its ongoing application in osteoporosis, a chronic condition enduring throughout life, with the underlying physiological process persisting [8].
When should denosumab be discontinued?	A treat-to-target approach has received more attention in recent years for the treatment of osteoporosis. T-score should be a key goal of treatment. Treatment with an anti-osteoporotic agent may be stopped if the T-score is above a value where there is a low risk of fracture to an individual. Nonetheless, it is recommended that if denosumab is stopped for any reason, therapy with bisphosphonates should be considered for sustained increased BMD[8].
What do guidelines recommend?	The Endocrine Society 2019 osteoporosis guidelines advised not to postpone or discontinue denosumab administration without the implementation of alternative therapy. Individuals at high risk should either persist with or transition to another therapy after a duration of 5 to 10 years. High-risk individuals should continue or switch therapy after 5 to 10 years. The European Calcified Tissue Society (ECTS) recommended that individuals at high risk of fracture, receiving denosumab for over 2.5 years should either persist with the treatment for up to 10 years or transition to zoledronic acid starting 6 months after the last denosumab injection, while closely monitoring bone turnover markers (BTMs). The American Association of Clinical Endocrinologists/American College of Endocrinology (AACE) 2020 suggests the continuation of denosumab for as long as it remains clinically appropriate [8].

## Conclusions

The evidence from the reviewed studies collectively suggests that denosumab is a promising therapeutic option for enhancing BMD, reducing fractures, and modulating BTMs in various populations, including postmenopausal women and men with low BMD as well as individuals with osteoporosis secondary to spinal cord injury. The consistent findings across different studies support the robustness of denosumab's efficacy and safety profile.

However, it's crucial to acknowledge some limitations in the review. The variability in study protocols, patient populations, and assessment methods introduces potential heterogeneity. The diverse patient characteristics may contribute to variations in treatment response, emphasizing the need for individualized considerations. Additionally, the relatively small sample sizes in certain studies warrant cautious interpretation, necessitating larger, well-controlled trials for more conclusive evidence. Large-scale randomized controlled trials with bisphosphonates are necessary to establish definitive evidence of the efficacy and safety of denosumab in treating osteoporosis in men.
